# Small supernumerary marker chromosomes – an update

**DOI:** 10.1186/1755-8166-7-S1-I11

**Published:** 2014-01-21

**Authors:** Thomas Liehr

**Affiliations:** 1 Jena University Hospital, Friedrich Schiller University, Institute of Human Genetics, Kollegiengasse 10, D-07743 Jena, Germany

## 

Genotype-phenotype correlations in patients with small supernumerary marker chromosomes (sSMC) are still difficult to asses.

The presently known influence of chromosomal imbalance induced by sSMC size and origin, mosaicism of sSMC in different cells of the body and uniparental disomy (UPD) of sSMC’s sister chromosomes on the clinical outcome is summarized according to data on ~5,000 sSMC cases summarized on http://www.fish.uniklinikum-jena.de/sSMC.html.

Two third of sSMC carriers are clinically normal. In the remainder 1/3 of sSMC patients, clinical symptoms may vary between slightly up to severely affected, including intrauterine death. Besides the known sSMC related syndromes Pallister-Killian-, isochromosome-15q12-, isochromosome-18p-, cat-eye- and Emanuel-syndrome there are numerous other yet unnamed and unidentified “sSMC-syndromes”. Recently, derivative-8- and derivative-13/21 syndromes in complex sSMC were reported.

The influence of chromosomal imbalance induced by sSMC size and its origin seems to have the largest impact on the phenotype of sSMC-patients. Besides UPD of sSMC’s sister chromosomes and mosaicism of sSMC may be important for the clinical outcome. The latter is especially important to be predicted in prenatal cases.

